# Rapid Range Expansion Is Not Restricted by Inbreeding in a Sexually Cannibalistic Spider

**DOI:** 10.1371/journal.pone.0095963

**Published:** 2014-04-23

**Authors:** Stefanie M. Zimmer, Henrik Krehenwinkel, Jutta M. Schneider

**Affiliations:** 1 Zoological Institute and Museum, Biozentrum Grindel, University of Hamburg, Hamburg, Germany; 2 Department of Evolutionary Genetics, Max Planck Institute for Evolutionary Biology, Plön, Germany; CNRS, France

## Abstract

Few studies investigated whether rapid range expansion is associated with an individual's short-term fitness costs due to an increased risk of inbred mating at the front of expansion. In mating systems with low male mating rates both sexes share potential inbreeding costs and general mechanisms to avoid or reduce these costs are expected. The spider *Argiope bruennichi* expanded its range recently and we asked whether rapid settlement of new sites exposes individuals to a risk of inbreeding. We sampled four geographically separated subpopulations, genotyped individuals, arranged matings and monitored hatching success. Hatching success was lowest in egg-sacs derived from sibling pairs and highest in egg-sacs derived from among-population crosses, while within-population crosses were intermediate. This indicates that inbreeding might affect hatching success in the wild. Unlike expected, differential hatching success of within- and among-population crosses did not correlate with genetic distance of mating pairs. In contrast, we found high genetic diversity based on 16 microsatellite markers and a fragment of the mitochondrial COI gene in all populations. Our results suggest that even a very recent settlement secures the presence of genetically different mating partners. This leads to costs of inbreeding since the population is not inbred.

## Introduction

Inbreeding, defined as the mating between two related individuals, increases the occurrence of homozygous deleterious alleles. The loss of heterozygosity leads to a decrease in the fitness of offspring, known as inbreeding depression [1] although inbreeding may also be associated with benefits [2]. Inbreeding depression has been reported for most taxa and has led to a variety of inbreeding-avoidance mechanisms [3] e.g. by sex differences in dispersal [4], [5], [6], in life-history [7], [8], or by mate choice [9]. The latter requires a kin-recognition mechanism (but see [10]) and can occur before but also after mating [11], [12]. Post-mating sexual selection requires multiple mating by females which increases copulation costs that should be offset, at least in part, by benefits [13]. Such benefits are particularly enigmatic if they are only of an indirect nature [14]. Indeed, avoidance or reduction of inbreeding costs through post-copulatory mate choice have been identified as a major benefit of female multiple mating in several taxa, such as house mice [15], birds [16], field crickets [17], [18], [19], and spiders [20].

In mating systems with classical sex roles (unselective males maximise fitness by increasing mating rates while reproductive success of females does not increase linearly with the number of mates [21]), females show a larger investment per offspring [22], [23] and suffer more from inbreeding through the loss of their individual fitness than males that only invested some sperm. Thus, selection to avoid inbreeding in the context of individual fitness costs should act particularly strong on females. However, in mating systems characterised by low male mating rates, males suffer similar fitness costs from inbreeding as females and avoidance of inbreeding should also be favoured in males. Indeed, when male mating rates are lower than female mating rates, selection should act more strongly on males than on females, particularly when polyandrous females possess means of cryptic female choice. These conditions are met in monogynous or bigynous mating systems, which are especially common in spiders [24], [25], [26], [27]. Males in such mating systems restrict themselves to mating with a single or maximally two females while females appear to favour multiple mating [26], [28]. It has been suggested that females oppose monopolisation by a single male through post-copulatory discrimination against less compatible males and there is some evidence that females cryptically discriminate against the sperm of related males [20]. However, to date no study has directly measured natural risks and costs of inbreeding for an individual in such mating systems.

Inbreeding is particularly likely if a small number of individuals split off from the original population and establish a new population representing only a fraction of the gene pool of the source population [29]. Furthermore, the co-settlement of siblings may promote the risk of inbreeding in the newly founded population. Analogous to the classical scenarios of founding populations and bottlenecks, although only short-term, species that actively expand their range will likely experience a decrease in genetic diversity at the forefront of range expansion in comparison to populations in the centre of a species' range [30]. This may result in an increased risk of inbreeding at least in the short term. Individuals that reproduce in a new patch may be faced with a reduced choice of mating partners that are perhaps even siblings. The lack of compatible mating partners can entail fitness costs as even one generation of inbreeding can lead to drastic fitness losses of the offspring, e.g. in terms of reduced competitive fertilisation success as reported for male *Telegryllus oceanicus* [31] or reduced adult lifespan in the spider *Argiope australis* (Welke & Schneider unpublished data). However, some species are tolerant to short-term inbreeding as for example *Stegodyphus lineatus* [6], *Oedothorax apicatus* [32] and *Anelosimus* cf. *jucundus* [33]. The degree of inbreeding depression can vary depending on the size and age of the mating population [34], as well as the potentially involved purging of deleterious recessive alleles [35], [36]. Generally, species that are successful colonisers are expected to show some tolerance towards the negative effects of inbreeding [37] or a dispersal mode that does ensure genetic diversity even in newly founded sites.

Here, we use the spider *Argiope bruennichi* (Araneae) that unites a mono- and bigynous mating system and has recently extended its range from southern Europe and Asia to Northern Europe [38]. The rapid colonisation implies that *A. bruennichi* can be considered a successful disperser. In combination with the observation that the species has started its range expansion from a large source population, it is likely that newly established populations even by small numbers of individuals encompass some genetic variation. *A. bruennichi* disperses aerially by ballooning and bridging to move within habitats. This passive mode of dispersal, particularly ballooning, entails a large component of chance as individuals can only influence direction by selecting certain wind conditions to fly [39], [40]. The expansion would likely occur through small numbers of individuals establishing new populations and as new meadows are colonised, individual females can expect high reproductive success. *A. bruennichi* spiderlings hatch simultaneously from large clutches after winter and likely disperse in groups from the same brood when conditions are favourable. This will lead to a situation in which many siblings from a single female are likely present in a meadow that also contains other families. Spiderlings may disperse short or long distances. This scenario creates both, inbreeding risk as siblings encounter one another and costs of inbreeding (note that costs of inbreeding require a population that is not inbred). Such a scenario match data derived from mating experiments and field observations that demonstrated selection to avoid the costs of inbreeding [20]. Hence, we predict that genetic diversity is present in small recently colonised meadows but that sibling matings will occur. As a consequence, we predict the presence of inbreeding depression from within-population matings, which should be absent in among-population matings. Hence, we expect a larger variation in hatching success resulting from the former matches in comparison from the latter ones and we expect this to be matched by the occurrence of sibling matches within populations.

We collected *A. bruennichi* egg-sacs and juveniles from four similar sized populations located near the northern edges of the species range. We assessed genetic diversity by analysing 16 microsatellite loci and a part of the mitochondrial COI gene. Furthermore, we assembled mating pairs that stemmed from the same egg-sac, from different egg-sacs of the same population or from two different populations and correlated the genetic distance of the mating partners with mating behaviour and hatching success. We predicted differences in mating behaviour with increasing relatedness of the mating partners and expected genetic distance to be positively correlated with hatching success.

While the sampled populations are all located within the recently colonised range of the species, they likely differ in their short-term settlement history in that they may have been populated early in the invasion process or in recent years.

## Material and Methods

### Study species

*Argiope bruennichi* [41] did not occur in Northern Europe until the beginning of the 20^th^ century with the exception of an isolated group around Berlin [42]. It expanded its range since around 1930 [38], [43] and colonised Northern Germany including the region around Hamburg since 1975 [43]. Today, these spiders are very common on meadows all over Northern Europe and can occur in densities of about 3 webs/m^2^ (Zimmer SM, personal observation).

As typical of entelegyne spiders, *A. bruennichi* possess paired mating organs. Females have two copulatory openings that are connected by two ducts to independent sperm storage organs (spermathecae) [44]. The two spermathecae can be filled separately by the same or two and rarely three males [45], [46]. Males have two secondary copulatory organs, the pedipalps, which they use to transfer their sperm. Because males damage their pedipalps during copulation, they can use both of them only once. The damaged genital part acts as a plug in the female's genital opening and is very effective in preventing rivals to mate into the same opening. This mechanism limits a female's mating rate [46].

Females show a highly aggressive mating behaviour. All females attack males during copulation and 80% of males are cannibalised by the female after mating [47]. These males have used only one of their paired pedipalps. Males that survive their first copulation may return and inseminate the second spermatheca of the same female or they may leave and search for a second mating partner [24]. All males inevitably die during their second copulation which can be found in other *Argiope* species as well [48], [49].

### Study Animals

We collected egg-sacs and juveniles from four geographically separated populations in the northern part of Germany (distance between population locations range between 42 and 148 km; Pevestorf (53°03′40.69″ N, 11°28′24.59″ E), Quarrendorf (53°15′51.81″ N, 10°01′30.74″ E), Buxtehude (53°27′10.37″ N, 9°40′23.67″ E), and Hamburg-Moorfleet (HH-Moorfleet; 53°30′37.30″ N, 10°6′1.60″ E)) between the end of April and the beginning of June 2010. There were no specific permissions required for these locations and the sampling did not involve endangered or protected species.

The collected egg-sacs were produced in 2009 and had overwintered. Several hundred spiderlings hatch out of the same egg-sac [50] and can hence be unambiguously labelled as siblings, although females may mate with two different males that share paternity so that spiderlings from the same egg-sac could be full or half-siblings [51]. The relatedness of juveniles could not be determined, so that these animals could not be used for sibling matings (see below).

387 individuals were raised from eggs in the laboratory until they reached adulthood. Each spider was individually labelled so that it was known from which population and from which egg-sac it derived. Males were kept in individual 250 ml plastic cups, whereas subadult females were housed in 330 ml plastic cups and were transferred in individual Perspex frames (36 _*_ 36 _*_ 6 cm) after they moulted to maturity. Mating trials were conducted in the frames, where females built their typical orb-webs. All spiders were sprayed with water five days a week. Males were fed with approx. 15 *Drosophila* spec., subadult and adult females with three *Calliphora* spec. on two days a week. After individuals' final moult, both females and males were weighed on an electronic balance (Mettler Toledo AB54-S) to the nearest 0.001 mg. All males and females used in the mating experiments were frozen at −80°C and preserved for genetic analyses (see below). Males were preserved after a single copulation and females were kept in the laboratory to produce egg-sacs until they died a natural death.

### Mating experiments

We experimentally staged and closely observed matings between siblings (N = 32), between non-siblings from the same population (N = 45) and between non-siblings from different populations (N = 62). Egg-sacs were collected from two populations (Buxtehude and HH-Moorfleet) so that we derived 15 maternal lines (8 from the population Buxtehude, 7 from the population HH-Moorfleet). Females and males from these matrilines were randomly assigned to one of the three mating trials. Spiders that were collected as juveniles were only used in the treatment where we arranged matings between different populations. 32 females from population HH-Moorfleet and Buxtehude were paired with males from the same family (sibling pairs; hatched from the same egg-sac); 45 females from population HH-Moorfleet and Buxtehude were paired with males from the same population that hatched out of a different egg-sac and 62 were mated to males that originated from different populations (HH-Moorfleet, Buxtehude, Quarrendorf, Pevestorf). Each mating pair was allowed a single copulation. Mating trials began by introducing the adult male into the frame threads of the female's web. Trials were terminated after the first copulation. If no mating occurred until one hour had passed, a new male was introduced to the female. A female was presented with a maximum of three males. It never happened that a female was not mated after introducing the third male. During every mating trial, we noted the times of male's first contact with the web and the female, the beginning and duration of courtship and copulation, the insemination duct the male copulated into and the occurrence of sexual cannibalism or male escape from a female attack.

### Hatching success

Mated females were transferred from the frame into 500 ml plastic cups where they built their egg-sacs. We obtained egg-sacs from 95 females, each of which produced 3.37±0.18 egg-sacs on average. All egg-sacs were weighed on the day of their construction and were visually inspected. Some egg-sacs were damaged or not completed. We selected all intact egg-sacs and left them to hatch. After the young had hatched from the eggs, egg-sacs were preserved and all eggs and spiderlings were counted under the microscope. Hatching success of all intact egg-sacs was determined by the following calculation: total number of spiderlings/((total number of eggs + spiderlings)/100).

### Microsatellite analysis and mitochondrial sequencing

We used microsatellite typing to estimate genetic distance (measured as the individual proportion of shared alleles; POSA) between individuals within and among the four populations of *A. bruennichi*. We were able to determine genetic distances in seven sibling pairs, 11 within-population pairs and in 31 among-population pairs.

For this, we extracted DNA with the 5 PRIME ArchivePure DNA Kit according to the manufacturer's protocol (5 PRIME, Hamburg, Germany).

We genotyped our specimens for a set of 16 previously developed microsatellite loci for *A. bruennichi* [42]. PCR amplification was performed according to the Qiagen Multiplex PCR Kit Protocol (see Qiagen, Hilden, Germany). We used ABI ROX size standard as size standard. Genotyping was performed on an Applied Biosystems 3730 DNA Analyzer. Microsatellite alleles were then called using GeneMapper 4.0 (Applied Biosystems). Genetic distances (POSA) within and among populations, as well as the overall F_ST_ value and pair-wise F_ST_ values among populations were calculated using Microsatellite Analyser (MSA) 4.05 [52]. Furthermore, we calculated heterozygosity of each individual and the allelic richness per population across the 16 microsatellite loci using MSA 4.05.

Due to the presence of null alleles (one or more alleles fail to amplify during PCR) for the microsatellite screened, detected with the software Microchecker 2.2.3 [53], we sequenced also a 1200 bp fragment of the mitochondrial COI gene as an additional marker. PCR and sequencing conditions are described in [42]. Sequences were edited using CodonCode Aligner (CodonCode Corperation, Centerville, USA) and aligned using ClustalW with default settings implemented in MEGA 4.0 [54]. Genetic Diversity (nucleotide and haplotype diversity) of the four populations was then calculated using DnaSP 5.10.1 [55].

### Statistics

Most data were analysed with the statistical program JMP 7.0.2. Non-normally distributed data (and residuals) were analysed with the non-parametric Kruskal-Wallis test. Significant differences between groups were specified with the Dunn test [56]. Tests of equal variances were performed with the Brown-Forsythe test. Linear or logistic regressions were used to test the influence of genetic distances on mating behaviour and hatching success. A multiple regression was used to test the influence of female's and male's heterozygosity on the hatching success of their offspring. All tests are indicated with the results. Descriptive statistics are given as mean ± standard error (SE). Sample sizes may differ between analyses due to missing data. Data are archived in Dryad: doi:10.5061/dryad.1np06.

## Results

### Hatching success

**Figure 1:**
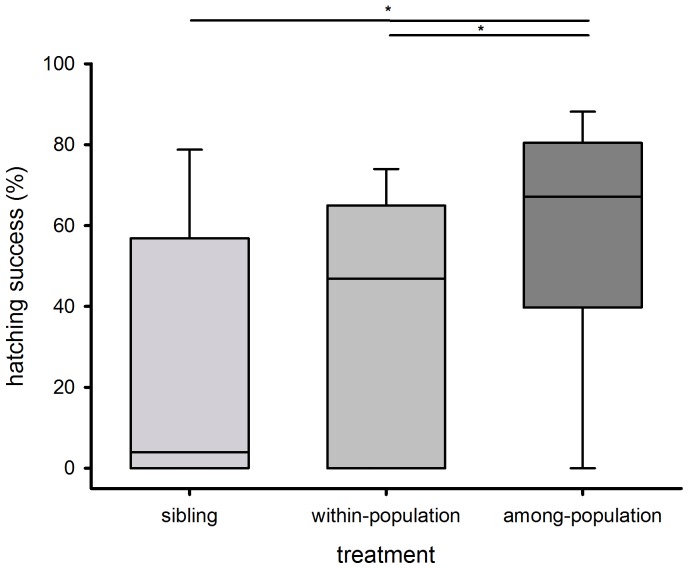
Hatching success (%) of the three mating treatments (sibling, within-population and among-population pairs). Hatching success was lowest for sibling matings (light grey), followed by within-population matings (grey) and was highest for among-population matings (dark grey). Box plots show the quartiles (box limits), the 10^th^ and 90^th^ percentiles (error bars) and the median (line). Statistically significant differences are indicated (*).

After a period of incubation, egg-sacs were opened and unhatched eggs and spiderlings were counted to determine hatching success. Hatching success was highly variable in all three treatments. As expected, the average hatching rate was lowest for sibling matings (28.18%±6.9; median = 3.95, N = 21), followed by within-population matings (40.63%±5.27; median = 46.84, N = 31) and was highest when the pair originated from different populations (57.0%±4.46; median = 67.15, N = 43; Kruskal-Wallis test: χ^2^ = 13.12, p = 0.0014; Figure 1 and Table 1). Multiple comparisons showed a significant difference of among-population and sibling groups (Dunn test; p = <0.01) as well as the among-population and within-population groups (Dunn test; p = <0.05); but comparisons between sibling groups and within-population groups were not statistically significant (Dunn test; p = >0.5; Figure 1). Variances in hatching success did not differ significantly between within-population and among-population matings (Brown-Forsythe test: F = 0.16, p = 0.69).

**Table 1:** Summarised results of the three mating treatments in *A. bruennichi* including averaged observed Heterozygosity (Ho) per female and male, averaged proportion of shared alleles (POSA) and averaged hatching success per mating pairs.

Treatment	Female H_o_	Male H_o_	POSA	Hatching success
sibling pairs	0.35±0.04 *(N = 8)	0.34±0.04 *(N = 9)	0.37±0.05 *(N = 7)	28.18±6.9 *(N = 21)
within-population pairs	0.36±0.05 *(N = 11)	0.33±0.03 *(N = 19)	0.61±0.03 *(N = 11)	40.63±5.27 *(N = 31)
among-population pairs	0.36±0.02 *(N = 33)	0.34±0.02 *(N = 36)	0.62±0.02 *(N = 31)	56.99±4.46 *(N = 43)

* Sample sizes may differ between results due to missing data.

### Genetic differences within and between source populations

The variability of all 16 microsatellite loci was high in all four source populations with a range from 5.6 to 6.4 numbers of alleles per locus referred to as allelic richness (Table 2).

**Table 2:** Summarised results for 16 microsatellite loci of four geographic different *A. bruennichi* populations including number of individuals per population (N), observed (Ho) and expected (He) heterozygosity per population, averaged proportion of shared alleles (POSA) per population as well as allelic richness (number of alleles per locus).

Population	N	Ho/He	POSA	Allelic richness	COI Nucleotide diversity*	COI Haplotype diversity*	No. of Haplotypes*
Hamburg-Moorfleet	65	0.38/0.62	0.58±0.003	6.1	0.00082	0.61299	5
Buxtehude	67	0.31/0.61	0.57±0.003	5.9	0.0015	0.70227	6
Quarrendorf	29	0.33/0.6	0.59±0.005	6.4	0.00113	0.68923	4
Pevestorf	31	0.36/0.56	0.53±0.005	5.6	0.00099	0.71077	5

* Nucleotide diversity and Haplotype diversity, as well as the number of Haplotypes of the four populations were calculated by the mitochondrial COI gene using DnaSP 5.10.1.

On average, all populations had a genetic distance between 0.5 and 0.6 (see Table 2). Comparison of the allelic richness among the four different populations across the 16 microsatellite loci did not reveal significant differences either (Kruskal-Wallis test: χ^2^ = 0.65, p = 0.89, N = 64). The observed heterozygosity of the four populations ranged from 0.31 to 0.38 and was much lower than the expected heterozygosity (range from 0.59 to 0.62; Table 2). Furthermore, the nucleotide diversity (range from 0.0008 to 0.0015) as well as the haplotype diversity (range from 0.61 to 0.71) of the four populations calculated by the mitochondrial COI data set showed similar genetic diversities within the four populations (Table 2). The number of haplotypes of the four populations ranged from 4 to 6 (Table 2). The overall F_ST_ value showed a moderate, but significant differentiation (0.052; p = 0.0001) and differentiation between all population pairs were significant (Table 3).

**Table 3:** Pair-wise F_ST_ -values (below diagonal) and the p-values (determined by permutation; above diagonal) for the four *A. bruennichi* populations based on 16 microsatellite loci.

	HH-Moorfleet	Buxtehude	Quarrendorf	Pevestorf
**HH-Moorfleet**		0.0001	0.0028	0.0001
**Buxtehude**	0.060975		0.0001	0.0001
**Quarrendorf**	0.025936	0.0401		0.0017
**Pevestorf**	0.056521	0.069954	0.021097	

### Genetic composition of pairs and mating behaviour

We pooled all mating pairs regardless of their origin and tested whether the number of shared alleles between female and male of a mating pair correlated with components of their mating behaviour. The duration of copulation (linear regression: F_1,47_ = 0.02, r^2^ = 0.0004, p = 0.9) and the frequency of cannibalism (logistic regression: χ^2^ = 1.85, p = 0.17, N = 49) were independent of the genetic distance between the mating partners. Furthermore, the genetic distance between a male and a female did not affect the time required until copulation occurred (linear regression: F_1,47_ = 0.02, r^2^ = 0.0004, p = 0.89).

### Genetic composition of pairs and hatching success

**Figure 2:**
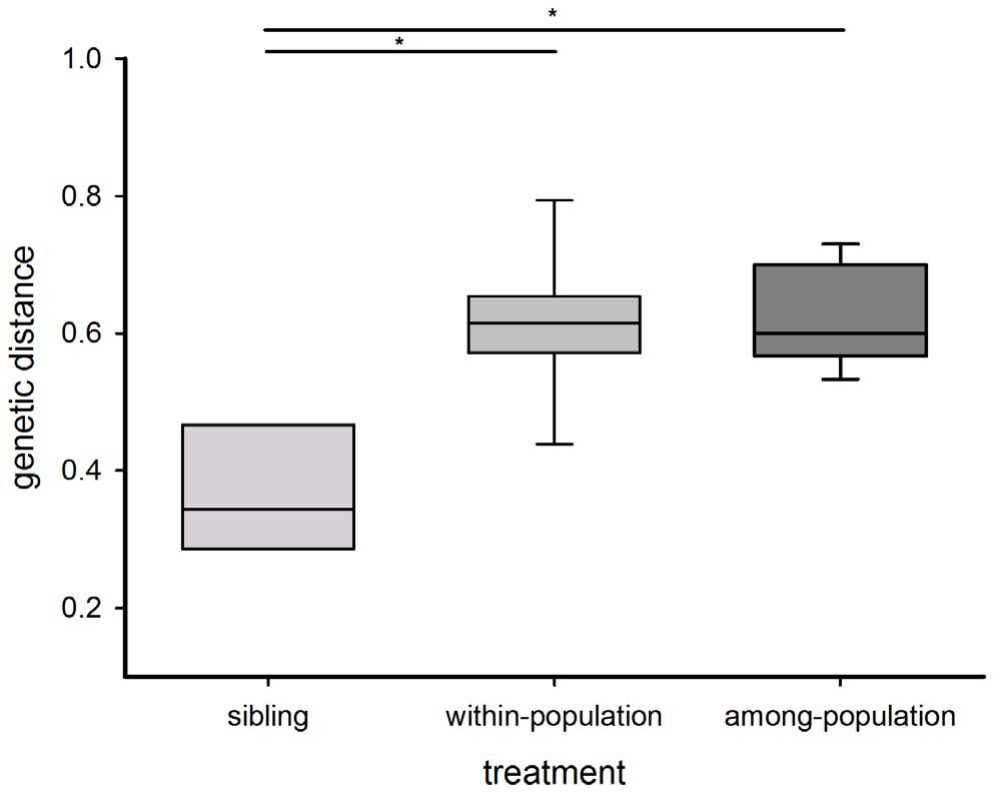
Genetic distance (POSA) of the three mating treatments (sibling, within-population and among-population pairs) measured as individual proportion of shared alleles. Box plots show the quartiles (box limits), the 10^th^ and 90^th^ percentiles (error bars) and the median (line). Statistically significant differences are indicated (*).

Comparing genetic distances among the three mating treatments, we expected to find the lowest genetic distance in sibling pairs, closely followed by a part of the within-population pairs while we expected the largest genetic distance in among-population pairs. A Kruskal-Wallis test showed significant differences of genetic distances between the three treatments (χ^2^ = 15.0, p = 0.0006, N = 49). However, we did not detect significant differences between unrelated pairs derived from the same (0.61±0.03) or from different populations (0.62±0.02; Dunn test; p = >0.2; Figure 2) while as expected, siblings had the lowest genetic distance (0.37±0.05; see Table 1) and significantly differed from the other two groups (Dunn test; p = <0.001). Variances in genetic distance did not differ significantly between the within-population and among-population groups (Brown-Forsythe test: F = 0.02, p = 0.88).

Using all mating pairs, the genetic distance between female and male of each pair did not significantly predict hatching success of their egg-sacs (linear regression: F_1,32_ = 1.28, r^2^ = 0.04, p = 0.27; Table 1). A multiple regression with the observed heterozygosity of female (F_1,33_ = −0.55, p = 0.58) and male of each pair (F_1,33_ = 0.79, p = 0.44) revealed no significant association with hatching success either (multiple regression: F_2,31_ = 0.37, r^2^ = 0.02, p = 0.69).

## Discussion

In experimental mating trials, we found the lowest hatching success in egg-sacs from pairs derived from the same brood and the highest hatching success when members of a pair came from different populations, while hatching success was intermediate for pairs of the same population. We genotyped each individual using 16 polymorphic microsatellite markers and expected that the presumed increase of genetic distance between the above groups of mating pairs would be mirrored in estimated proportion of shared alleles. However, while our measures of genetic distance provided expected estimates for siblings, we neither detected differences in genetic diversity between our study populations nor could we relate reduced hatching success in clutches derived from within-population matings to genetic distance between mating pairs. There are two possible explanations for the inconclusive mismatch between genetic and reproductive data. Either reduced hatching success in within-population matings was not caused by inbreeding depression or our genetic markers alone were not appropriate to detect relatedness between pairs.

The low hatching success of egg-sacs from sibling pairs strongly suggests that the species would suffer from inbreeding depression if sibling matings did occur. Studies of other species found much lower costs of sibling matings in terms of fitness traits such as hatching success, fecundity and survival [6], [32] and significant negative effects were apparent after only three generations of inbreeding. It was suggested that spiders might show a high tolerance towards inbreeding, perhaps as an adaptation to cope with a relatively high incidence of sibling matings [6]. Our data imply a comparatively low tolerance to inbreeding in *A. bruennichi*, but also a low risk of inbreeding even in small, recently founded populations. Genetic diversity was high in all populations and was probably even underestimated as the sampling mostly occurred before a possible ballooning event.

Within-population matings resulted in an intermediate hatching success with a very high variation and the variation in genetic distance was also highest in this group. This corroborates our predicted scenario and may suggest that some pairings were distinctly less profitable than others while the majority matched well. By coincidence, the majority of pairings in this treatment may have used offspring from unrelated females. The high variance may tentatively suggest that there is a possibility of less compatible matings if spiders stayed close to their birth site. The design of this study may have not been sufficient to detect the actual probability of sibling matings. It is possible that such matings can only be estimated by investigating small-scale spatial patterning of individuals as it has been measured in e.g. insects [57], [58]. To date, we have no data on the within-population sub-structuring on a scale relevant for mating and distance covered by males during mate search in *A. bruennichi*. Therefore, we cannot accurately estimate the probability for individuals of encountering a sibling.

Generally, a loss in genetic variation would be expected in any species that colonises new habitats as most dispersal mechanisms will result in a small number of individuals that found new populations and hence only represent a subset of the genetic variation of the source population [29]. Spiders lay their eggs in large clutches and egg-sacs of *A. bruennichi* contain several hundreds of eggs [50]. In species with an overwintering period such as *A. bruennichi*, all egg-sacs in a population hatch very synchronously regardless of when they were produced [59]. The common dispersal mode after hatching in spiders is ballooning, which means that the animal releases a thread of silk until it is uplifted by thermic or wind [60]. This mode of travelling is generally restricted to very small spiders and is risky since the spider has very limited options to control where it will be going [39], [40]. Hence one might expect that at least a proportion of hatchlings remain at their natal site, which has proven to be of sufficient quality. These spiders may disperse by walking or bridging and settle nearby, causing a population substructure with patches of individuals that are closely related. Such a pattern has been found in the eresid spiders *Stegodyphus lineatus* [6] and *S. tentoriicola* [61], in which newly established nests are clustered around maternal sites. Unless there is sex-specific early dispersal, males may mature in the proximity of their sisters promoting inbreeding. In *S. lineatus*, males initially mate close to their birth site accepting a risk of inbreeding and then adopt a long distance mate search of higher risk [6]. Furthermore, a few spider females can produce a lot of offspring and quickly fill suitable web-sites at a location with her offspring. Depending on the degree of substructure and the probability of mating with a sibling, selection should favour kin-recognition mechanisms during mate choice if inbreeding is associated with more costs than benefits. However, generally rejecting related individuals as mating partners can be disadvantageous if the probability of finding a different mating partner is unpredictable. Female web-building spiders do not actively search for mates and face a risk of remaining unmated, hence they may benefit from accepting any male initially to secure fertilisation of her eggs leaving options for further copulations with preferred sires. Polyandry will then be in the female's interest because paternity could be biased towards the best mate [14], [62]. Post-copulatory choice has been demonstrated in several *Argiope* species [63], [64] and it was shown to be based on relatedness in *A. lobata* in which females cryptically favour sperm from unrelated males [20]. Pre-copulatory recognition seems to be present as well, since siblings mate for shorter and have a lower rate of sexual cannibalism [65]. Such a strategy enables males that survive their first copulation to leave and search for a better second mating opportunity [66]. A trading-up mechanism, in which both, females and males, first mate indiscriminately to secure a sperm supply and then try to re-mate with a higher quality mate, appears to be relatively common in spiders [20].

While the above conditions largely apply for *A. bruennichi*, the high variation in all our samples strongly suggests that dispersal is very efficient in this species so that each patch of suitable habitat will soon be inhabited by a relatively large number of individuals from several origins [42].

Even though our treatment of mating individuals that originated from the same population showed a reduced hatching success, this effect was not apparent in the genetic distance of the experimental pairs. Several authors suggest that a sufficient number of markers are required to detect inbreeding depression in natural populations [67], [68]. Even studies with a relatively large number of microsatellite loci (>20) gave poor evidence for inbreeding depression [68]. By using 16 polymorphic microsatellite loci we clearly detected the difference between siblings and non-siblings, but no differences within the latter group added to the notion that such measurements alone are not always appropriate to predict risks and costs of inbreeding. The reduced hatching success of within-population matches might have resulted from incompatibilities that are not detected using microsatellites.

Indeed, we found an amino acid change between Alanine and Threonine in the mitochondrial genome of several individuals. It seems that pairs in which females carry this mutation and mated with males from a different population exhibit a higher hatching success (unpublished data). The interaction between the mutation and genetic composition of mating pairs suggests more complex genetic interactions and might be one possible explanation of the higher reproductive success of among-population pairs compared to the within-population pairs.

It remains an open question how relevant incompatible matings are in natural populations that may show a much larger intermixture of genotypes through long distance dispersal. The rapid range expansion of *A. bruennichi* suggests that they are potent ballooners although it is unclear whether all hatchlings of an egg-sac balloon or whether a proportion stays. Published accounts are inconsistent in this respect [69], [70]. One would expect that an obligate high-risk dispersal phase should be opposed by selection just as much as the opposite of no dispersal, which would facilitate inbreeding as well as kin competition.

As the calculation of heterozygosity based on the microsatellite data set revealed a conspicuous difference between observed and expected heterozygosity of the four populations that did not relate to the genetic distance data, the presence of null alleles was tested for each locus and was confirmed in some loci. Null alleles occur through a failure of amplification during PCR leading to an over-estimation of homozygotes. Therefore we chose the mitochondrial COI gene as an additional marker to better trace the genetic diversity of the four populations. However, a DnaSP analysis of the COI gene data confirmed the similar genetic diversity within the four populations. A comparison with other studies showed that null alleles seem to be widespread in spiders [71], [72]. This might be explained by enormous population sizes of spiders providing increased mutation opportunities that lead to changes in primer binding sites and consequently inaccurate sequencing with the designed microsatellite primers. Moreover, in *A. bruennichi* an admixture of different lineages occurs resulting in the introgression of Asian alleles in populations of Northern Europe [42] that might lead to an excess of non-amplifying loci. Future studies on spiders that involve usage of microsatellite markers should be aware of a potentially high risk of null alleles.

In conclusion, our results show that sibling matings lead to severe inbreeding depression in *A. bruennichi* spiders and that there should be strong selection for inbreeding avoidance. The genetic data suggest that active partner choice would be beneficial even in small and recently founded populations as the genetic diversity is high and consequently the probability of finding a compatible partner is generally high. However, reduced hatching success in pairings of spiders derived from egg-sacs of the same population was not mirrored in the genetic distance data. Incompatibilities other than those caused by inbreeding may be responsible for the reduced hatching success.

Due to the experimental exclusion of ballooning and missing data on small scale population sub-structuring in *A. bruennichi*, the probability for individuals of encountering siblings cannot yet be estimated accurately. Studies are under way to close this gap by identifying the genetic population structure of natural populations close to and during the mating season on a small spatial scale. Furthermore, future experiments are of interest to test whether *A. bruennichi* has evolved pre-copulatory avoidance mechanisms to prevent or at least reduce costs of inbreeding depression in the field. During field studies, we commonly observed that males reject virgin females in the field without any obvious reasons [51], [73]. Incompatibilities that result in reduced hatching success might be a reason.
